# Influence of Altitude on Phytochemical Composition of Hemp Inflorescence: A Metabolomic Approach

**DOI:** 10.3390/molecules25061381

**Published:** 2020-03-18

**Authors:** Luca Giupponi, Valeria Leoni, Radmila Pavlovic, Annamaria Giorgi

**Affiliations:** 1 Centre of Applied Studies for the Sustainable Management and Protection of Mountain Areas (CRC Ge.S.Di.Mont.), University of Milan, Via Morino 8, 25048 Edolo (BS), Italy; luca.giupponi@unimi.it (L.G.); valeria.leoni@unimi.it (V.L.); anna.giorgi@unimi.it (A.G.); 2 Department of Agricultural and Environmental Sciences—Production, Landscape, Agroenergy (DISAA), Via Celoria 2, 20133 Milan, Italy

**Keywords:** hemp, cannabinoids, terpenes, altitude, high-resolution mass spectrometry

## Abstract

The phytochemical profiling of hemp inflorescences of clonal plants growing in different conditions related to altitude was investigated. Four strains of industrial hemp (*Cannabis sativa* L., family Cannabaceae) of Kompolti variety were selected and cloned to provide genetically uniform material for analyses of secondary metabolites (terpenes, cannabinoids, and flavonoids) at two different elevations: mountain (Alagna Valsesia 1200 m ASL) and plains (Vercelli Province 130 m ASL). Environmental conditions influenced by elevation have proven to be important factors inducing variations in hemp inflorescences’ secondary metabolite composition. In fact, all plants grown at altitude exhibited a higher total amount of terpenes when compared with plains counterparts, with β-Myrcene, *trans*-Caryophyllene and α-Humulene as the main contributors. A metabolomic, un-targeted approach performed by HPLC-Q-Exactive-Orbitrap*^®^*-MS platform with subsequent data processing performed by Compound Discoverer™ software, was crucial for the appropriate recognition of many metabolites, clearly distinguishing mountain from plains specimens. Cannabidiolic acid CBDA was the most abundant phytocannabinoid, with significantly higher concentrations in the mountain samples. The metabolic pathway of CBGA (considered as the progenitor/precursor of all cannabinoids) was also activated towards the production of CBCA, which occurs in considerably 3 times higher quantities than in the clones grown at high altitude. Isoprenoid flavones (Cannaflavins A, B, and C) were correspondingly upregulated in mountain samples, while apigenin turned out to be more abundant in plains samples. The possibility to use hemp inflorescences in pharmaceutical/nutraceutical applications opens new challenges to understand how hemp crops respond in terms of secondary metabolite production in various environments. In this regard, our results with the applied analytical strategy may constitute an effective way of phytochemical profiling hemp inflorescences.

## 1. Introduction

Hemp (cannabis, *Cannabis sativa* L., family: Cannabaceae) was frequently cultivated in the past, but its agronomic expansion was interrupted in the beginning of the 1950s for many reasons, one of them being the presence of psychoactive substance Δ-9-tetrahydrocannabinol (THC) that is produced by some hemp varieties. Nowadays, this has been partly obliterated and the European Union permits the cultivation of hemp with THC content of less than 0.20% [1]. For example, in Italy, regulation n°242/2017 [2], delineates the conditions for hemp production, its commercialization, and utilization for specific industrial purposes. Therefore, different genotypes have been registered, along with standardized cultivation procedures [3]. Many ecologically, agronomically, and pharmaceutically positive properties qualify this multifunctional crop as an opportune raw material for various traditional (fiber, food, oil, medicine) or innovative industrial applications (new biomaterials and biofuels) [4,5]. 

The European Union has regulated commercial production and distribution of more than 70 hemp varieties [6], among which the Kompolti variety is one of the oldest. This hemp variety is frequently cultivated in continental European countries. This well-known dioecious variety was developed in Hungary as the first hybrid breed [7] in order to produce seeds for oil production, but also its stalks have been largely exploited. The particularity of this strain is its growing dynamics: it needs a whopping 6-month flowering period that usually finishes in October. Its macro/microscopical botanical aspects, geographical distribution, and agricultural status have been comprehensively studied, but surprisingly, no information is currently available on the content of the high added-value bioactive substances that are characteristic of its flowers. 

The *C. sativa* inflorescence contains a number of chemically active compounds, such as cannabinoids, terpenoids, and flavonoids. The most important secondary metabolites are phytocannabinoids whose acidic forms are exclusively biosynthesized in the glandular trichomes, which are abundant on the surface of the female flowers [8]. Inflorescences of industrial hemp varieties, Kompolti included, are particularly rich in cannabidiolic acid (CBDA) that is disposed to the spontaneous decarboxylation to cannabidiol (CBD) under favorable environmental/conservational circumstances such as warming and light [9]. CBD is responsible for a variety of pharmacological actions that may have some remarkable applications, but unlike THC, CBD does not possess any psychoactive effects [10]. CBD has been studied intensively over the past decade due to its biomedical relevance. Several studies suggest that CBD can be effective in treating epilepsy and other neuropsychiatric disorders, including anxiety and schizophrenia [11]. This is the reason why CBD dietary supplements obtained from different industrial *C. sativa* chemotypes have become particularly widespread [12]. On the other hand, it is important to evaluate the main factors that determine CBD production in hemp inflorescence—if it depends on the genetic predisposition of hemp chemovar, or its production is conditioned by the environment, in particularly the geographical position where plant is bred [13].

Although CBD and THC are the crucial phytocannabinoids, hemp trichomes themselves are capable of generating a whole series of acidic/decarboxylated phytocannabinoids: about 120 have been isolated to date [8]. Based on their appearance in the metabolic pathway that involves their formation, all phytocannabinoids are categorized into 11 subclasses [9], where the central position belongs to cannabigerolic acid that is synthesized from geranyl diphosphate and olivetolic acid [14]. CBGA further provides tetrahydrocannabinolic acid (THCA), CBDA, and cannabichromenic acid (CBCA). Cannabigerol (CBG), THC, CBD, and cannabichromene (CBC) are corresponding neutral equivalents. Other phytocannabinoids detected in hemp inflorescences comprise main oxidation products of THCA and CBDA: cannabinol (CBN) and cannabinolic acid (CBNA) obtained from THC(A), and cannabielsoin (CBE) and cannabielsoinic acid that derive from CBDA. The “cannabivarin” group, commonly following the above-mentioned ones, is produced from condensation of geranyl diphosphate with divarinic acid, which results in a propyl instead of the pentyl side-chain. 

Even though the attention of the scientific community has been focused on major phytocannabinoids, the phytochemical characterization of *C. sativa* highlights the presence of various non-cannabinoids constituents including flavonoids [15]. Their characterization is scarce and random, especially when the inflorescences of industrial hemp are concerned [13,16,17]. In any case, the characterization of the cannaflavones—compounds isolated exclusively from hemp—needs further elucidation, mainly when the inflorescences are concerned. 

One non-phytocannabinoid category of bioactive secondary metabolites that is studied in much more detail is the terpene family. They represent volatile components that has been claimed to have a synergic action with cannabinoids [18]. Many different monoterpenes and sesquiterpenes are important components of *C. sativa* essential oils [19,20], as they define some of the unique organoleptic properties and may also influence nutraceutical potentials of different hemp strains and varieties [21].

Selecting a genotype appropriate for a particular end-use application that is adaptable to an environment is of principal importance for successful hemp cultivation. Hemp is a plant adaptable to various growing and ecological conditions, but there are no data available in the literature that concern the differences that may arise from cultivating the same chemovar contemporarily in the plains and mountain habitats. Different hemp varieties cultivated at high altitudes showed a characteristic phytochemical and ecological behavior [13].

In this research, our aim was to study whether two very different ecological environments (mountain and plains) would have a significant impact on phytocannabinoids qualitative and quantitative content, flavonoids presence, and terpenoids profile, in order to study the plant phytochemical behavior and its potential to provide nutraceutical substances.

Four strains of industrial hemp (Kompolti) were selected and cloned to provide genetically uniform material for analyses of secondary metabolites (cannabinoids, terpenes, and flavonoids) of clones of the same plant grown at different elevation in two sites representative of lowland (Vercelli Province 130 m ASL) and mountain (Alagna Valsesia 1200 m ASL) during the growing season 2018.

## 2. Results and Discussion

Multi-targeted applications of industrial hemp with the environmental benefits related to its cultivation, have raised interest in its production. Special attention has been paid to *C. sativa* inflorescences that represent a promising added-value product with remarkable pharmacological and nutraceutical effects [13,16,22,23]. The phytochemical composition of inflorescences has been studied intensively, but there is not substantial information that regards the differences that may rise due to geographical/microclimate factors. In this experiment, raw inflorescences material obtained from plants cultivated in the Italian Alps at two different elevations was evaluated. Mountain (M) region was located in the commonality of Alagna Valsesia (1200 m ASL), whereas plains (P) cultivation was performed in the Province of Vercelli (130 m ASL). 

### 2.1. Terpenoids Profile Estimated by HS-SPME- GC-MS Analytical Procedure

Monoterpenes, diterpenes, triterpenes, and sesquiterpenes are important components of the *C. sativa* resin responsible for its unique aromatic properties [20,21]. Considering the terpenoids fraction characterized by high-volatile features, the headspace solid-phase microextraction-gas chromatographic mass-spectrometric (HS-SPME-GC-MS) analytical approach presents the best methodology for their comprehensive profiling, as was confirmed by our recently published studies [12,24,25]. SPME is a simple and fast modern tool used to characterize the volatile fraction of secondary metabolites of different parts of plants [26,27] and animals [28].

Complete data concerning the terpenes fingerprint in the two groups of Kompolti inflorescences are summarized and reported in Table 1 It was possible to define 20 compounds that belong to the mono/di/tri terpenes and 21 sesquiterpenes. Our results are qualitatively comparable with those reported by others [19,20,22,29]. The most remarkable aspect is that four M clones expressed significantly higher amounts of both terpenoids subgroups, with high variation in the individual quantitative profile. This is the reason why the results are elaborated by statistical approach that consisted of the comparation of relative amounts of each compound between mountain and corresponding plain clone. Generally, the predominant monoterpene was β-myrcene, followed by both α-/β-pinene and limonene, although without uniformity between four plants (Table 1). For example, the plant M2 was particularly rich in β-myrcene, followed by β-pinene and limonene, but not α-pinene. On the other hand, the plant 1 (both M and P samples) expressed its specificity in the accumulation of β-ocimene, while others were very poor in its presence. Also, plant 1 contained the oxygenated terpene 4,8-epoxy-p-menth-1-ene that was completely absent from other inflorescences. 

Geographic origin, accompanied by environmental conditions, turned out to be an important variable that determined the sesquiterpenes’ quantitative characteristics: mountain plants exhibited higher total amounts than plains counterparts, with trans-caryophyllene and α-humulene as the main contributors (Table 1). Those two compounds are typical constituents of *C. sativa* essential oil [30], and also here showed a quite stable relevance. Selina-3,7(11)-diene was detected in a moderate amount in all mountain samples, but its presence was not detected in two plains clones. α-ylangene, α-bergamotene and β-farnesene expressed highly inconsistent trends. For example, P1 samples were particularly abundant in β-farnesene, while in plant 3 (both P and M samples) it was absent. All samples contained longicyclene, the sesquiterpene rarely identified in *C. sativa* inflorescences, with the exception of one drug-type chemovar [31].

In all cases, the qualitative and quantitative differences observed in the chemical profile of terpene fractions were conditioned by many factors such as, hemp variety, cultivation and environmental conditions, harvest time and post-harvest conditions, storage and drying of raw plant, and extraction procedure applied. Despite the fact that within each group, plants were grown under identical conditions and treated in the same way, it remains an open question why each of them had its specific terpenoids fingerprint.

### 2.2. Quantification of Cannabinoids by HPLC-Q-Exactive-Orbitrap^®^-MS Analysis

Based on the available commercial cannabinoids standards, quantification of inflorescence extracts was performed by applying our validated method [13,24,25], as explained in detail in the material and methods section. Quantitative data related to the analysis of the content of phytocannabinoids in the two inflorescences groups performed by means of the HPLC high-resolution mass spectrometry (HRMS, Q-Exactive-Orbitrap^®^-MS) are shown in Table 2. The quantification was performed for CBD (cannabidiol), Δ^9^-THC (delta-9- tetrahydrocannabinol), CBN (cannabinol), CBC (cannabichromene), CBG (cannabigerol), CBDV (cannabidivarin), Δ^9^-THCV (delta-9-tetrahydrocannabivarin) and the acid forms CBDA (cannabidiolic acid), Δ^9^-THCA (delta-9- tetrahydrocannabinolic acid), CBNA (cannabinolic acid), CBCA (cannabichromenic acid), CBGA (cannabigerolic acid), CBDVA (cannabidivarin acid) and Δ^9^-THCVA (delta-9-tetrahydrocannabivarin acid). In contrast to what was found for volatile terpenoids, the results obtained for cannabinoids were more uniform with respect to the cultivation site. It was therefore feasible to perform paired statistical evaluations combining the specimens from both locations. Since the Kompolti chemovar belongs to the fiber-type hemp, it is not surprising that CBDA was the most abundant phytocannabinoid with significantly higher concentration in the mountain than in the plains. Those values (both for M and P group) are higher than those recently reported by [32,33]. The THC and THCA content were below legal limits, but the occurrence of traces of CBNA —THCA’s non-enzymatic, oxidative product—should be taken into consideration when so-called “total THC” amount is concerned. Furthermore, it is noted that the metabolic pathway of CBGA (considered as the progenitor/precursor of all cannabinoids) was activated towards the production of CBCA, which occurred in considerably 3 times higher quantities in the samples cultivated at high altitude. 

The presence of similar quantities of CBD in the mountain and plains specimens indicated that geographical location did not significantly influence decarboxylation of CBDA. This process naturally occurs under the action of heat and light, but here it is more probable that it was caused by Kompolti’s predisposition to have prolonged flowering in the late summer/beginning autumn season, when the average daily temperatures are moderately higher in both locations. Therefore, a genetically predisposed flowering season may have led to a partial conversion of the parent CBDA into its neutral counterpart, as was demonstrated for Futura and Finola 75 varieties [13].

### 2.3. HPLC-Q-Exactive-Orbitrap^®^-MS Untargeted Metabolomics Approach: Phytocannabinoids Profiling and Identification of Polyphenolic Structures 

**Figure 1:**
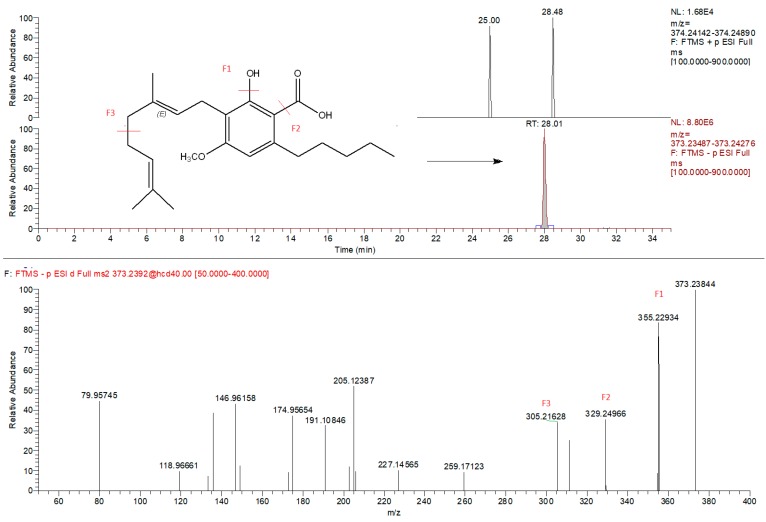
Extracted ion chromatogram (*m/z* = 373.2384) of CBGMA (cannabigerolic acid methyl ester) and corresponding MS/MS spectra identified exclusively in negative mode.

**Figure 2:**
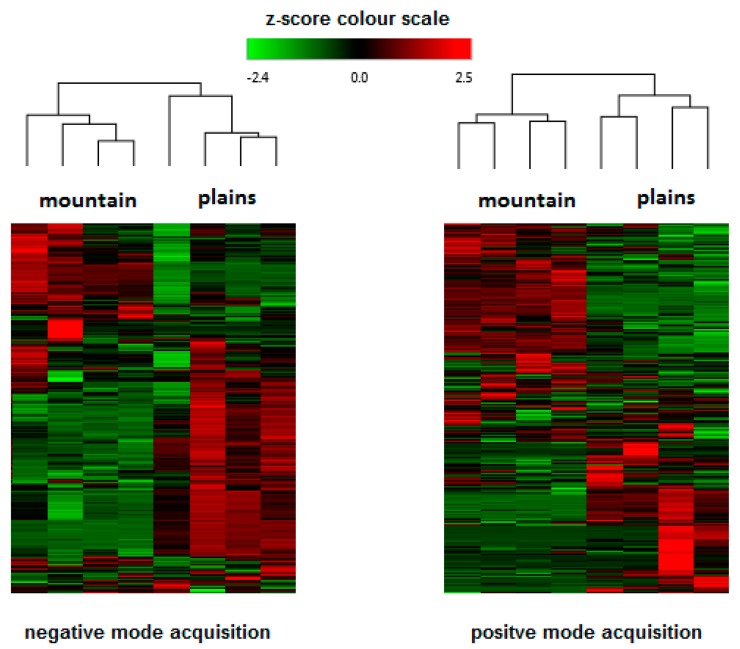
Hierarchical cluster analysis: heat-map reflecting the differences between compounds revealed in Kompolti inflorescences in respect to different geographical/climatic conditions.

Metabolomic fingerprinting of the inflorescences that can be used for pharmacological/nutraceutical purposes is important to evaluate a plant metabolite quality and variability. Chromatographic/HRMS fingerprints have been used in the modelling and prediction of pharmacological activities of many medicinal plants [34], but only sporadically for cannabis. Also in this study, as performed in our recently published work [13] the compounds that characterize hemp inflorescences were identified by HPLC-Q-Exactive-Orbitrap^®^-MS untargeted metabolomics approach that consist of chromatographic separation, HRMS acquisition, and post-analysis data elaboration applying the Compound Discoverer software. Unlike in the above-mentioned paper, here we analyzed the samples separately, using two types of ionization: both positive and negative (Table 3) that enabled a more profound approach. The negative mode revealed the presence of about 80 compounds, whereas the positive mode individuated more than 190. By introducing the detection in negative polarity, it was possible to identify compounds not detected previously [13]. For example, CBGA methyl ester (CBGMA) was not detected in positive ionization, but its presence was clearly confirmed by fragmentation pattern obtained in negative mode (Figure 1). This strategy consequently allowed for an in-depth cluster analysis that clearly demonstrated the differences between the samples coming from the mountains and those cultivated in the plains (Figure 2). Two acquisition modes showed to be complementary in the statistical evaluation of differences between two samples group. As regards the data obtained, it was possible to detect other minor secondary metabolites already identified in other cannabis species. The CBD-family remains to be most abundant (Table 3), enriched by the presence of two sesquiterpene CBDA esters. This CBDA-terpene (inter)reaction needs further elucidation, especially whether it depends on climate/environmental conditions [8,9,13].

In any case, special attention must be paid to cannaflavins that belong to the class of prenylflavonoids. This flavins are secondary metabolites exclusive to the *Cannabis* genus and were detected in both environments. Their notable presence in mountain samples points towards alternations in their synthesis [16]. The higher levels detected in the mountain samples may possibly be a consequence of the lower average temperature, combined with high solar radiation experienced at the beginning of plant flowering. In fact, it was reported that different classes of flavonoids are involved in plant protection mechanisms, specifically for their radical scavenger activity and screening ability against short wavelength UV-B light [35].

**Figure 3:**
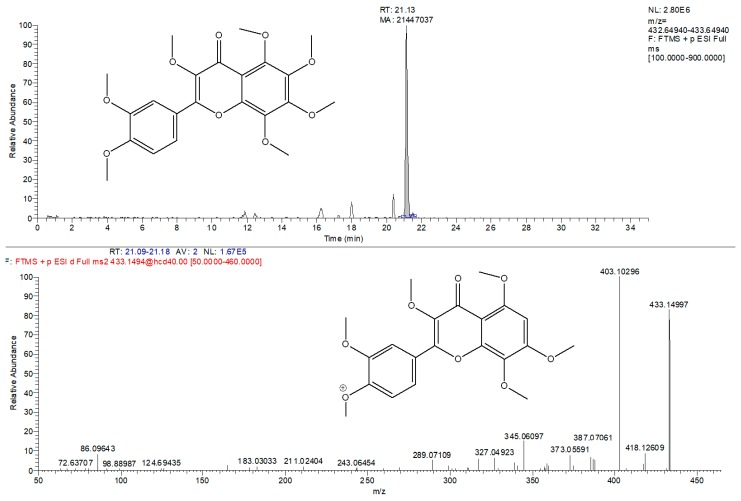
Tentative identification of 3-methoxynobiletin. Extracted ion chromatogram (*m/z* = 433.1493) and corresponding MS/MS spectra.

Furthermore, in the Kompolti inflorescence, the remarkable presence of a signal with m/z value at 433.14931 was identified and leads towards the recognition of a particular flavonoid: 3-methoxinobiletin (3,3′,4′,5,6,7,8-heptamethoxyflavone) (Figure 3). This compound has not been reported for any variety of hemp so far. Furthermore, it belongs to the class of polymethoxyflavones that have anti-inflammatory and anti-carcinogenic activities and occur as “novel nutraceutical compounds” [36]. In order to perform its absolute identification, it is necessary to isolate it from the inflorescence and define it with a more detailed analytical approach, also including NMR analysis.

Our metabolomics mapping identified two important phytohormones: salicylic and abscisic acid that have not been reported for cannabinoids inflorescences so far. Their presence was confirmed in our mountain samples, but their amount turned to be more than 10 times higher in lowland specimens. These findings are important to highlight because of the fact that salicylic acid was identified as ‘calorigen’, the plant hormone that induces heat-production in some inflorescences [37]. Also, salicylic acid plays a critical role in the defense against biotrophic pathogens and in the response of plants to abiotic stress, predominantly drought, temperature, heavy metals and, osmotic stress [38]. As far as abscisic acid is concerned, it is well known that stress conditions affect its endogenous production and catabolism rates, while exogenously applied abscisic acid influenced the content and biosynthesis of terpenoids in *C. sativa* [39].

## 3. Materials and Methods

### 3.1. Experimental Fields and Samples Collection

#### 3.1.1. Clones

Seedlings of *C. sativa* were grown starting from commercial certified seeds of the Kompolti variety. In a 120-plant seedling tray, the most vital plants were chosen for cloning to provide material of genetic homogeneity. Four pairs of clone plants were chosen to grow at altitude versus in the plains, assigning them a number indicating a single plant strain (1,2,3,4) and a letter for the growing site (M for ones destined to be grown at altitude and P for ones destined to be grown in the plains). These strains included only low Δ 9-THC fiber strains. Plants were grown potted in a loam-vermiculite-sand mixture (6:2:1) under ambient greenhouse conditions. Cuttings were taken from the parent pistillate plant of each strain, treated with Rootone, and rooted in perlite. The standard soil permitted to avoid pedotrophic variability. Then, the three-week rooted plants potted in the same substrate were transported in the mountain location (municipality: Alagna Valsesia; elevation 1200 m ASL; latitude 45°51′ N; longitude 7°56′ E) and a lowland location (municipality: Vercelli 130 m ASL; latitude 45°19′ N; longitude 8°22′ E). The geographical area is located in Piedmonte, north of Italy, in the Western Alps ecoregional section for what concerns the mountain location, with prevailing temperate semi-continental bioclimates and in the Po Plain ecoregional section, with prevailing temperate subcontinental bioclimate for what concerns the lowland experimental station [40].

#### 3.1.2. Plant Parts Sampled

Harvest of inflorescences was carried out at flowering, corresponding to the phenological codes 2202 [41]. It was considered “inflorescence” only the 15 cm upper part of the stem. The sectioned parts of the inflorescences were left to air-dry, protected from light in open containers at room temperature (25 °C) for 2 weeks [42]. They were subsequently preserved in plastic bags under vacuum stored in a cool room until analysis. The low temperature avoided changes in metabolites, cannabinoids, and terpenes. The inflorescences material of each clone was sampled five times to realize the analyses. Then, the extracts were injected three times in the analytical instruments. 

### 3.2. Chemical and Reagents 

For head-space (HS) analysis, the SPME coating fiber (DVB/CAR/PDMS, 50/30 µm) was obtained from Supelco (Bellefonte, PA, USA) while acetonitrile, 2-propanol, formic acid (all LC-MS grade) were purchased from Carlo Erba (Milan, Italy). Ultrapure water was obtained through a Milli-Q system (Millipore, Merck KGaA, Darmstadt, Germany). All cannabinoids were analytical standards at concentration 1mg/mL (methanolic solution) were purchased from Sigma Aldrich, Round Rock, Texas).

### 3.3. Superfine Grinding (SFG) Sample Preparation

Samples (1.0 g each) were transformed in fine powder in a high intensity planetary mill at a frequency of 25 Hz for 1 min, using two 50 mL jars (precooled with liquid nitrogen) with 20 mm stainless steel balls. 

### 3.4. Accelerated Solvent Extraction (ASE) for Cannabinoids Profiling 

The extraction procedure was done according to the our already-published procedure [12,24,25]. In brief, all extractions were performed by accelerated solvent extraction apparatus using an ASE 350 (Thermo-Fisher Scientific, Waltham, MA, USA) with 34-mL stain steel cells. Inflorescence powder (100 mg) was mixed with an equal weight of diatomaceous earth and transferred into the cell. One-hundred μL of solution containing the IS (diazepam 1 mg/mL) was added and cell was filled with diatomaceous earth. ASE operation parameters were as following: room temperature of 25 °C, pressure (1500 psi), number of static cycles (2 cycles, 5 min each), purging time (60 s with nitrogen) and rinse volume (90%). Extracts (25 mL) obtained using pure methanol and were dried under vacuum; the residue was dissolved in 1 mL of acetonitrile. The resultant solution was diluted (1:10) in starting mobile phase, 2 μL were submitted to analysis by HPLC-Q-Exactive-Orbitrap-MS. Commercially available officinal plants mixture previously analyzed for the absences of cannabinoids served as blank samples and were used to obtain the matrix-matched calibration curves. Matrix-matched calibration curves were obtained by spiking the standard solutions of 14 commercially available cannabinoids that covered the two-concentration range: 0.1 to 10 μg/g and 10–1000 μg g^−1^. 

### 3.5. Cannabinoids HPLC-Q-Exactive-Orbitrap-MS Evaluation 

The cannabinoids profile was assessed employing the method recently published by us [13,24]. HPLC-Q-Exactive-Orbitrap*^®^*-MS analysis was achieved on an HPLC Surveyor MS quaternary pump, a Surveyor AS autosampler with a column oven, and a Rheodyne valve with a 20-μL loop system (Thermo Fisher Scientific, San Jose, CA, USA) using a reverse-phase HPLC column 150 × 2 mm i.d., 4 μm, Synergi Hydro RP, with a 4 × 3 mm i.d. C18 guard column (Phenomenex, Torrance, CA, USA). The mobile phase consisted of water and acetonitrile gradient both acidified with 0.1% formic acid. The gradient (flow 0.3 mL/min) started with 95% of 0.1% aqueous formic acid with a linear decrease up to 5% in 30 min. The mobile phase was returned to initial conditions at 35 min, followed by a 5-min re-equilibration period. The column and sample temperatures were 30 °C and 5 °C, respectively. The mass spectrometer Thermo Q-Exactive Plus (Thermo Scientific, San Jose, CA, USA) was equipped with a heated electrospray ionization (HESI) source. Capillary temperature and vaporizer temperature were set at 330 and 380 °C, respectively, while the electrospray voltage was set at 3.30 kV. Sheath and auxiliary gas were 35 and 15 arbitrary units, with S lens RF level of 60. The mass spectrometer was controlled by Xcalibur 3.0 software (Thermo Fisher Scientific, San Jose, CA, USA. Qual Browser in Xcalibur 3.0 software) was used for the exact mass and isotopic pattern determination. The FS-dd-MS^2^ (full scan data-dependent acquisition) in positive and negative mode was used for both screening and quantification purposes. Resolving power of FS adjusted on 70,000 FWHM at *m/z* 200, with scan range of *m/z* 100–900. Automatic gain control (AGC) was set at 3e^6^, with an injection time of 200 ms. A targeted MS/MS (dd-MS^2^) analysis operated in both positive and negative mode at 35,000 FWHM (*m/z* 200). The AGC target was programmed at 2e^5^, and maximum injection time was set at 100 ms. Fragmentation of precursors was optimized as three-stepped normalized collision energy (NCE) (20, 40 and 40 eV). Detection was based on retention time and on calculated exact mass of the protonated/deprotonated molecular ions, accompanied with fragmentation pattern [13].

### 3.6. HPLC-Q-Exactive-Orbitrap-MS Untargeted Metabolomics Approach 

Raw data from high resolution mass spectrometry were elaborated with Compound Discoverer™ (Thermo Scientific), that facilitated the peak recognition, retention times arrangement, profile assignment, and isotope pattern [43]. Metabolite identification was based on accurate mass and mass fragmentation pattern spectra against MS-MS spectra of compounds available on mzCloud database (HighChem LLC, Slovakia, https://www.mzcloud.org) and in the literature [44]. The ChemSpider Web services (https://www.chemspider.com) and Human Metabolome platform (https://hmdb.ca/) was used as supplementary confirmation tools. If mass fragmentation pattern did not correspond to any of databases annotated by Compound Discoverer™ software, manual confirmation of their fragments using program ChemDrow was completed. 

### 3.7. HS-SPME and GC-MS Analysis for Terpenes Examination

Complete analytical technique was provided in detail in our recently published article [12,24,25]. In brief, 100 mg of inflorescence powder was put into 20 mL glass vials along with 100 μL of the IS (4-metil-2-pentanone, 20 mg/mL in 2-propanol). A cap with a silicon/PTFE septum (Supelco, Bellefonte, PA, USA) was used to close the vial, which was then kept in the temperature block (37 °C), (CTC Analytics, Zwingen, Switzerland). At the end of the sample equilibration time (30 min), a conditioned (60 min at 280 °C) SPME fiber was subjected to the sample for 120 min using a CombiPAL system injector autosampler (CTC Analytics, Zwingen, Switzerland). 

Analyses were performed with a Trace GC Ultra coupled to a Trace DSQII quadrupole mass spectrometer (MS) (Thermo-Fisher Scientific, Waltham, MA, USA) equipped with an Rtx-Wax column (30 m × 0.25 mm i.d. × 0.25 µm film thickness) (Restek, Bellefonte, PA, USA). The oven temperature program was: from 35 °C, held for 8 min, to 60 °C at 4 °C/min, then from 60 to 160 °C at 6 °C/min and finally from 160 to 200 at 20 °C/min. Helium was the carrier gas, at a flow rate of 1 mL/min. The MS was operated in electron impact (EI) ionization mode at 70 eV, *m/z* range of 35–350. An alkanes mixture (C8-C22, Sigma R 8769, Saint Louis, MO, USA) was run under the same chromatographic conditions as the samples to calculate the Kovats Retention Indices (RI) of the detected compounds [27,45]. Compounds were recognized by comparing with authentic standards or by using the Kovats retention indices in combination with the literature and via the National Institute of Standards and Technology (NIST) MS spectral database. The semi-quantitative evaluation was achieved using the internal standard procedure and the results were expressed as µg/g IS equivalents. 

### 3.8. Statistical Analysis

The relative intensity of chromatographic peaks was processed by Compound Discoverer platform that enabled Hierarchical Cluster Analysis. Differences between two groups were evaluated using a two-tailed Student’s *t*-test from the BioVinci statistical program (Version 1.1.4., BioTuring, Inc. 2018 California, CA, USA). A *p*-value of less than 0.05 was deemed statistically significant.

## 4. Conclusions

The quantity and quality of secondary plant metabolites are often attributed to a combination of genetic and environmental factors. Eliminating genetic and pedotrophic factors, the results accomplished in this study indicate qualitatively and quantitatively intraspecific variations in secondary metabolites, other than a major effect attributable to the ecological conditions related to the elevation of the location. A mountain environment, with condition of UV length exposure and critical conditions, deeply influences the quantity of the inflorescence compounds, favoring the production of CBDA and cannaflavins. Information regarding the differences in industrial hemp inflorescences phytochemical profile supports hemp cultivation in mountain areas as a source of pharmacologically active cannabinoids, terpenes and cannaflavones that are considered also as promising nutraceuticals. Metabolomics approaches delineated this crop as resourceful and highly adaptable to the variation of climate/geographical conditions.

## Figures and Tables

**Table 1:** Mono/di/triterpenes and sesquiterpenes extracted and identified by headspace solid-phase microextraction-gas chromatographic mass-spectrometry (HS-SPME-GC/MS) in mountain (M) and plains (P) inflorescences.

**MONO/DI/TRI Terpenes**	**M1**		**P1**		***p*** **Value ^b^**	**M2**		**P2**		***p*** **Value**	**M3**		**P3**		***p*** **Value**	**M4**		**P4**		***p*** **Value**
	**mean ^a^**	**± SD**	**mean**	**± SD**		**mean**	**± SD**	**mean**	**± SD**		**mean**	**± SD**	**mean**	**± SD**		**mean**	**± SD**	**mean**	**± SD**	
α-Pinene	3823.9	81.1	2705.8	152.3	0.014	2401.3	56.3	4659.2	394.3	0.013	4870.9	3.7	1944.8	65.0	<0.001	1788.6	190.1	1560.3	89.1	n.s.
α-Fenchene	n.d.	n.d.	26.3	3.0	<0.001	44.4	27.6	n.d.	n.d.	<0.001	n.d.	n.d.	13.1	1.1	<0.001	n.d.	n.d.	12.4	2.6	<0.001
Camphene	202.6	4.7	139.1	5.2	0.008	991.6	6.8	270.2	10.3	<0.001	292.5	5.3	91.0	1.1	<0.001	162.0	20.1	121.6	0.2	0.004
β-Pinene	2074.0	152.8	2067.3	149.8	0.050	12965.6	55.6	2979.8	233.7	<0.001	2258.6	20.7	1058.5	94.8	0.0015	1456.0	336.7	1230.8	3.0	n.s.
β-Myrcene	26,294.5	450.3	23745.1	1070.4	0.020	76,993.1	4716.5	16,893.5	519.5	0.0008	23,597.9	716.7	5949.9	24.1	0.0002	26,723.6	2988.9	11,660.5	1778.3	0.004
Limonene	2603.1	112.5	3023.2	150.3	n.s.	11472.7	276.0	4184.1	134.1	<0.001	5565.0	846.0	962.2	1.4	0.011	5538.9	93.8	3883.5	8.0	<0.001
β-Phellandrene	642.8	6.2	628.5	40.6	n.s.	2017.8	10.1	515.9	34.6	0.0012	662.0	348.3	188.5	2.2	0.0015	681.6	54.9	417.5	20.4	0.026
*Cis*-ocimene	352.70	1.6	321.5	60.3	n.s.	132.0	2.2	38.8	4.5	<0.001	44.6	1.0	8.6	0.7	<0.001	34.4	9.8	34.5	3.2	n.s.
γ-Terpinene	37.0	5.9	23.3	7.6	0.002	86.5	1.0	77.6	0.4	0.004	69.0	10.9	49.9	1.4	0.054	18.5	5.3	15.6	0.8	n.s.
β-Ocimene	6571.3	25.5	5555.3	40.1	0.001	135.20	5.1	138.0	2.0	n.s.	493.7	16.3	28.9	28.9	<0.001	139.6	6.7	110.4	6.3	0.001
α-Terpinolene	136.4	15.4	143.1	26.3	n.s.	486.1	48.4	190.2	41.2	0.0002	274.3	7.7	45.4	0.9	<0.001	192.0	6.5	144.6	10.3	0.002
Terpene	6.0	0.1	4.8	0.1	0.007	12.8	0.9	5.3	0.7	<0.001	4.5	0.2	1.2	0.1	<0.001	11.4	0.4	0.8	0.0	<0.001
α-Fenchone	47.5	2.6	20.0	7.0	0.009	84.2	28.2	74.6	24.6	0.045	74.3	14.3	9.4	0.6	0.017	41.1	0.5	36.6	1.6	0.020
Alloocimene	80.9	0.1	91.7	17.1	n.s.	37.6	4.1	24.2	1.2	0.015	12.1	4.1	2.2	1.2	<0.001	23.3	8.3	17.2	6.2	0.036
Linalyl oxide	19.6	0.7	16.2	2.1	0.070	8.5	0.1	7.2	5.3	0.040	18.7	1.2	17.4	1.7	n.s.	21.1	0.8	16.6	1.6	n.s.
4,8-Epoxy-p-menth-1-ene	147.8	16.8	167.9	13.0	0.012	n.d.	n.d.	n.d.	n.d.	-	n.d.	n.d.	n.d.	n.d.	-	n.d.	n.d.	n.d.	n.d.	-
Pinalol	117.0	1.6	130.0	20.0	n.s.	271.2	2.1	269.6	11.7	n.s.	248.2	31.2	229.8	20.2	0.1	139.8	12.4	131.2	5.9	0.1
β-Linalool	149.6	7.7	163.0	49.5	n.s.	693.6	3.4	576.4	31.3	0.028	617.0	49.8	54.9	2.4	<0.001	1118.5	94.2	880.7	8.6	0.010
α-Fenchol	68.0	43.0	29.0	10.0	n.s.	164.6	48.2	130.8	35.9	n.s.	368.7	36.6	12.2	2.3	0.0014	171.9	71.6	12.7	2.7	0.060
Verbenol	89.8	36.4	37.7	18.9	0.035	204.5	155.1	65.1	0.6	n.s.	212.1	159.9	2.4	0.5	n.s.	6.8	6.5	11.7	1.3	n.s.
tot	43,464.5		39,038.8			109,203.3		31,100.5			40,141.9		11,038.4			38,269.1		20,299.2		
**Sesquiterpenes**	**M1**		**P1**		***p*** **Value**	**M2**		**P2**		***p*** **Value**	**M3**		**P3**		***p*** **Value**	**M4**		**P4**		***p*** **Value**
	**mean**	± SD	**mean**	**± SD**		**mean**	**± SD**	**mean**	**± SD**		**mean**	**± SD**	**mean**	**± SD**		**mean**	**± SD**	**mean**	**± SD**	
α-Ylangene	130.8	7.3	201	19	0.044	47.1	13.7	60.7	6.6	0.05	15.6	6.5	3.4	0.9	0.007	13.3	6.5	9.2	0.2	n.s.
α-Copaene	41.7	1.2	50.7	4	n.s.	21.9	0.1	48.8	14.6	n.s.	9.7	0.6	1.9	0.2	0.002	22.1	2.3	20.8	0.8	0.027
Zingiberene	62.6	6.4	106.8	10.5	0.02	68.3	6.8	41.7	11.2	<0.001	26	0.6	35	12.3	0.081	122.7	11.7	111.8	4.9	n.s.
Longicyclene	314.2	80.9	417.5	59.9	0.09	44.2	0.7	25	17.2	n.s.	43	0.9	2.5	0.8	<0.001	302.6	63.2	207.1	11.2	0.08
α-Bergamotene	976.9	58.1	1440.9	115.3	n.s.	831.8	310.9	381.8	49.2	n.s.	326.5	54.1	77.9	13.9	0.004	3140.1	204.7	2395.8	86.2	0.008
*Trans*-Caryophyllene	6487.1	113.7	3668.7	502	0.008	9206.9	144.9	3797.7	41.8	0.007	3345.5	456.2	829.2	28.2	<0.001	7017.4	922.6	1684.6	38.2	<0.001
Aristolene	55.8	3.6	105	9.8	0.005	18.4	0.7	28	10.3	n.s.	2.63	2.63	0.1	0.91	n.s.	n.d.	819	114.4	0	<0.001
Isoledene	33.2	6.9	67.3	10.9	0.004	31.9	11.9	31.8	10	n.s.	8.4	4.8	2.69	1.8	n.s.	13.4	1.1	12.7	0.4	n.s.
β-Santalene	12.3	2.2	23.9	3.7	0.005	6.9	0.2	7	2.5	n.s.	4.1	3.15	0.38	0.21	n.s.	8.2	1.3	10.7	2.5	n.s.
Aromadendrene	152	40.2	76.3	9.3	n.s.	195.7	1.4	7.6	0.1	<0.001	19.9	1.4	14.3	2.8	n.s.	22.4	1.4	2.8	0.3	0.001
α-Humulene	3190	15	2099	183.2	0.007	3107.1	177.4	1284.2	9.9	0.002	3206.1	204.5	306.2	15.8	0.014	3431.4	87.2	2660.4	100.2	<0.001
β-Farnesene	51.9	41.3	1495.4	4.6	<0.001	1105.7	14.3	249.6	59.6	0.002	n.d.	n.d.	n.d.	n.d.	-	1494.2	17.4	1244.8	13.4	0.006
β-Selinene	416.4	29.7	632.4	49.2	0.003	245.5	47.2	502.8	50.4	<0.001	123.2	1.9	211.7	10.6	0.003	112.6	28.5	271.5	7.6	0.004
α-Selinene	260.8	11.9	379.9	46.2	0.027	61.8	6.4	145.4	35.1	0.034	4.9	0.4	23.3	6.1	0.033	74.5	4.2	71.2	8.7	n.s.
β-Bisabolene	739.6	30.4	293.7	156.9	0.05	1515.6	29.5	581.2	21.5	<0.001	557	51.2	129.9	8.8	0.003	1278.2	8.9	1024.2	14.3	0.003
α-Farnesene	213.8	29.6	357.2	68.4	0.045	661.2	21.3	314.2	14.4	0.008	160.8	26.6	37.9	21.2	0.031	500.2	18.2	583.2	86.2	n.s.
δ-Cadinene	128.2	20.8	215.1	38.5	n.s.	63.5	4.2	74.2	4.6	0.013	124.4	0.2	23.9	3.7	0.008	66.8	5.2	33.4	20.2	n.s.
β-Maaliene	657.2	25.9	1048	224.2	0.008	297.8	12.4	197.2	10.2	0.0078	63.3	10.5	59.6	14.1	n.s.	354.2	25.1	362	2.7	n.s.
Selina-3,7(11)-diene	1821	134.4	n.d.	n.d.	<0.001	1623.3	157.2	n.d.	n.d.	<0.001	581.6	81.6	132.5	12.6	0.007	1196.2	25.2	992.3	102	n.s.
Caryophyllene oxide	59.2	6.3	63.2	32	n.s.	68.2	6.9	30.1	4.3	0.024	18.9	4.1	6	0.7	0.006	67.2	2.4	73.6	4.6	n.s.
Guaiol	114.5	20.9	144.8	48.2	n.s.	326.2	28.1	280.4	70.7	n.s.	214.4	56.6	337	20.2	n.s.	289.2	15.4	227.9	8.6	n.s.
10-Epi-γ-Eudesmol	329.6	26.7	170.7	47.2	0.003	425.2	42.4	331.2	3.1	0.004	243.2	51.4	42.5	0.1	0.005	181.1	21	295.2	3.1	n.s.
tot	16,248.8		13,057.5			19,974.2		8420.6			9099.2		2277.9			19,708.0		12,409.6		

^a^ Data are given as mean ± SD (standard deviation), *n* = 3 (expressed as µg/g SI equivalents). ^b^ *p*-value—*t*-test with 95% two-tailed confidence interval for difference of means.

**Table 2:** Results regarding the bioaccumulation of the main cannabinoids in the inflorescences of mountain Kompolti samples and corresponding plains clones (µg/g, mean of four biological samples ± SD).

	Mountain		Plains		Statistical Significance
	Mean	SD (±)	Mean	SD (±)	
Neutral forms					
CBD	5300	3500	6000	3800	ns
Δ^9^-THC	<LOQ	/	<LOQ	/	/
CBN	<LOQ	/	<LOQ	/	/
CBC	460	120	120	50	0.005
CBG	110	10	180	80	<0.001
CBDV	250	400	450	40	<0.001
Δ^9^-THCV	<LOQ	/	<LOQ	/	/
Acid forms					
CBDA	99,600	24,800	68,220	15,000	0.01
Δ^9^-THCA	840	200	1010	400	ns
CBNA	40	4	50	10	ns
CBCA	1570	200	570	30	0.008
CBGA	7410	900	4510	400	0.015
CBDVA	310	70	240	20	ns
Δ^9^- THCVA	<LOQ	/	<LOQ	/	/

LOQ–limit of quantification 1 µg/g for all phytocannabinoids. ns: not significative

**Table 3:** Results regarding metabolomic identification in Kompolti inflorescences.

Class	Compound	Formula	(M + H)^+^/Main Fragment	(M − H)^−^/Main Fragment	RegulationMountain vs. Pains
*Phytocannabinoids*					
CBG cannabigerol class	CBG	C_21_H_32_O_2_	317.2475/193.1223	315.2329/191.1078	Upregulated in mountain
	Sesqui-CBG	C_26_H_40_O_2_	385.3173/193.1223	n.i.	
	6,7-epoxy-CBG	C_21_H_32_O_3_	333.2424/315.1867	n.i.	
	CBGVA	C_20_H_27_O_4_	333.2060/173.0962	331.1915/313.1809	
	6,7-epoxy-CBGA	C_22_H_32_O_5_	377.2323/341.2113	375.2185/257.3077	
	CBGA	C_22_H_31_O_4_	361.2375/219.1017	359.2228/191.1078	
	CBGMA	C_23_H_34_O_4_	n.i.	373.2384/355.2293	
	Sesqui-CBGA	C_27_H_40_O_4_	n.i.	427.2854/409.2748	
CBD (cannabidiol) class	CBDV	C_19_H_26_O_2_	287.2006/165.0914	285.1860/217.1234	Upregulated in mountain
	Nor-CBD	C_20_H_28_O_2_	301.2162/179.1070	299.2017/231.1391	
	CBD	C_21_H_30_O_2_	315.2319/193.1223	313.2173/191.1078	
	CBDM	C_22_H_32_O_2_	329.2475/229.0812	327.2329/205.1234	
	CBDVA	C_20_H_26_O_4_	331.1904/313.1801	329.1758/217.1123	
	Nor-CBDA	C_20_H_28_O_4_	345.2060/327.1956	343.1915/231.1391	
	CBDA	C_22_H_30_O_4_	359.2219/341.2114	357.2017/245.1547	
	CBDMA	C_32_H_46_O_4_	n.i.	371.2228/259.1704	
	Sesquiterpene-CBDA ester	C_32_H_46_O_4_	495.3469/341.2114	493.3323/357.2017	
	γ-Eudesmyl-CBDA ester	C_37_H_54_O_4_	562.4017/341.2114	561.3949/357.2017	
Δ^9^-THC tetrahydrocannabinol class	THCV	C_19_H_26_O_2_	287.2006/165.0914	285.1860/217.1234	ns
	THC	C_21_H_30_O_2_	315.2319/193.1223	313.2173/n.i.	
	THCVA	C_20_H_26_O_4_	331.1904/313.1801	329.1758/189.0921	
	THCA	C_22_H_30_O_4_	359.2219/341.2114	357.2071/245.1547	
CBC cannabichromene class	CBCV	C_19_H_26_O_2_	287.2006/165.0914	285.1860/163.0765	Upregulated in mountain
	CBC	C_21_H_30_O_2_	315.2319/193.1223	313.2173/n.d.	
	CBCVA	C_20_H_26_O_4_	331.1904/313.1801	329.1758/189.0921	
	CBCA	C_22_H_30_O_4_	359.2219/341.2114	357.2071/313.2179	
CBN cannabinol class	CBN	C_21_H_26_O_2_	311.2007/223.1118	309.1860/n.i.	ns
	CBNA	C_22_H_26_O_4_	355.1904	337.1800	
	Cannaflavin A	C_26_H_28_O_6_	437.1964/313.0709	435.1813/309.0413	
Isoprenoid flavones	Cannaflavin B	C_21_H_20_O_6_	369.1333/313.0706	367.1195/309.0499	Upregulated in mountain
	Cannaflavin C	C_26_H_28_O_6_	437.1964/313.0709	435.1813/309.0414	
Polymethoxyflavones	3-Methoxynobiletin	C_22_H_24_O_9_	433.14980/403.10296	n.i.	Upregulated in mountain
Flavones	apigenin	C_15_H_10_O_5_	271.0601/nd	269.0455/117.0348	Upregulated in plains
Phenolic acid	Salicylic acid	C_7_H_6_O_3_	n.i.	137.0426/95.8554	Upregulated in plains
	Abscisic acid	C_15_H_20_O_4_	n.i.	263.1289/219.1391	

(M + H)^+^: exact mass of pseudomolecular ion acquired in full scan positive ionization mod; (M − H)^−^: exact mass of pseudomolecular ion acquired in full scan negative ionization mode; main fragment: the base fragment in MS/MS spectrum; ns: not significant differences between two chemovars; A: acid; V: C3 chain length; Nor: C4 side chain length; M: methyl ester; n.i.: not identified; Regulation: hierarchical cluster analysis (Figure 3).
